# Thermal transformation of polar into less-polar ginsenosides through demalonylation and deglycosylation in extracts from ginseng pulp

**DOI:** 10.1038/s41598-021-81079-w

**Published:** 2021-01-15

**Authors:** Fan Yao, Xiang Li, Jing Sun, Xinxin Cao, Mengmeng Liu, Yuanhang Li, Yujun Liu

**Affiliations:** National Engineering Laboratory for Tree Breeding, College of Biological Sciences and Biotechnology, Beijing Forestry University, Beijing, 100083 China

**Keywords:** Natural products, Mass spectrometry, Liquid chromatography

## Abstract

The present study was conducted to qualitatively and quantitatively elucidate dynamic changes of ginsenosides in ginseng pulp steamed under different temperatures (100 or 120 °C) for different durations (1–6 h) through UPLC-QTOF-MS/MS and HPLC with the aid of as numerous as 18 authentic standards of ginsenosides. Results show that levels of eight polar ginsenosides (i.e., Rg_1_, Re, Rb_1_, Rc, Rb_2_, Rb_3_, F_1_, and Rd) declined but those of 10 less-polar ginsenosides [i.e., Rf, Rg_2_, 20(*S*)-Rh_1_, 20(*R*)-Rg_2_, F_4_, 20(*S*)-Rg_3_, 20(*R*)-Rg_3_, PPT, Rg_5_, and 20(*R*)-Rh_2_] elevated with increases of both steaming temperature and duration; the optimum steaming conditions for achieving the highest total ginsenosides were 100 °C for 1 h. Particular, 20(*R*)-Rg_3_, a representative less-polar ginsenoside with high bioactivity such as potent anti-cancer effect, increased sharply but Re, the most abundant polar ginsenoside in fresh ginseng pulp, decreased dramatically. More importantly, ginsenoside species enhanced from 18 to 42 after steaming, mainly due to transformation of polar into less-polar ginsenosides. Furthermore, four malonyl-ginsenosides were detected in fresh ginseng pulps and ten acetyl-ginsenosides were formed during steaming, demonstrating that demalonylation and acetylation of ginsenosides were the dominant underling mechanisms for transformation of polar into less-polar ginsenosides.

## Introduction

Ginseng (*Panax* ginseng C.A. Meyer) is a perennial plant belonging to the family Araliaceae. It has long been utilized as a functional food (natural tonic) or medicinal plant in Korea, Japan, and China for more than 2000 years^1^, and nowadays is widely known all over the world due to its multiple health-promoting and pharmacological functions such as anti-cancer, anti-fatigue, antioxidant and anti-aging^2^. The main bioactive compositions of *P*. ginseng and several other *Panax* species are triterpene saponins, termed ginsenosides, which are considered to be responsible for a variety of their pharmacological actions^3^.

Until now, more than 150 ginsenosides, usually but not always written as R ‘x’, have been identified. The ‘x’ is determined by the distance of movement of a certain ginsenoside on a thin-layer chromatography plate, with the most polar one being marked as ‘A’ whereas the least polar one as ‘H’^4,5^. Based on their backbone structures, ginsenosides are divided into four groups, i.e., protopanaxadiols with a dammarane backbone, protopanaxatriols with an additional hydroxyl group at C-6 on a dammarane backbone, oleanolic acids with a pentacyclic triterpenoid base, and those of an ocotillol type with a five-membered epoxy ring at C-20. Numerous varieties of sugar molecules are attached to different positions of the backbone, forming a diversity of further more ginsenosides, including Ra_1-3_ and Rb_1-2_ (five protopanaxadiols), Re and Rf (two protopanaxatriols), Ro (an oleanolic acid) and Rs (an ocotillol)^5^. Although not all, numerous ginsenosides have pharmacological effects. For instances, Rh_1_, Rg_3_, Rb_1_, compound K, Rg_5_, and Rg_1_ have been demonstrated clearly to inhibit inflammatory responses by suppressing activities of various inflammasomes, including the NLRP3 and NLRP1 (NLRP, nucleotide-binding oligomerization domain-like receptor protein), and AIM2 (absent in melanoma 2 inflammasomes)^6,7^. Several ginsenosides such as Rg_1_, Rk_1_ and Rg_5_ have also been attested to protect against acetaminophen-induced liver injury^8–10^. Furthermore, Kang et al.^11^ found that both 20(*R*)-Rg_3_ and 20(*S*)-Rg_3_ inhibited lytic replication and viral proliferation of the MHV-68 (murine herpesvirus 68), and the two Rg_3_ isomers also efficiently repressed chemically-induced lytic replication of human gamma herpesviruses in both EBV-positive BC-3 and KSHV-positive Raji cell lines.

Ginseng root is commercially available as white and red ginsengs. White ginseng is produced by dehydration of the fresh ginseng either under direct sunlight, in the shade or under the light bulb, and red ginseng is manufactured by steaming the fresh ginseng first at 95–100 °C for 2–3 h, then dried under sunlight^12,13^. Previous reports suggest that red ginseng holds more potent anticancer activities and higher bioactive potential than white ginseng does^14,15^. The differences in biological effects of white and red ginsengs are attributed to a significant transformation of ginsenosides during steaming. There is a general acceptance of such a view, and to a large extent it has also been confirmed, that less-polar ginsenosides which are rarely present in white ginseng possess stronger bioactivity, and their contents and species increase while those of polar ginsenosides decrease upon steaming^16^. Similar conclusion has also been drawn from studies on steamed and non-steamed American ginseng^17^.

In Traditional Chinese Medicine, ginseng root, the most commonly used part of the herb, is normally harvested from field ginseng plants at the age between 5 and 10 years old. However, ginseng flower and berry generated since its third or fourth year can be collected more than once, are produced only as byproducts and, are even abandoned as residue after washing off from ginseng seed. In our previous study, it was observed that the ginsenosides content of ginseng flower enhanced and their ginsenosides species changed both dramatically after steaming^16^. Studies further confirmed that ginseng flower and pulp (the flesh portion of the ginseng berry) possess profiles of ginsenosides that are substantially different from those of ginseng root^16,18^. In particular, ginseng pulp contains high levels of the ginsenoside Re and total ginsenosides, amounting roughly seven and four times as those in ginseng root, respectively^19^. Luo et al.^20^ reported that steaming of American ginseng pulp augmented content of the ginsenoside Rg_3_ and enhanced anti-proliferative effects toward two human colorectal cancer cell lines. Nevertheless, no report has been dealt with changes in ginsenosides composition by focusing on mechanism of ginsenosides conversion before and after steaming treatments on ginseng pulp.

The ongoing development in ultrahigh-performance liquid chromatography (UPLC) coupled with various MS-based metabolomics exhibits advantages of high resolution, selectivity and sensitivity, thus can be employed in rapid analyses of components from complex medicinal herb mixtures^13^. Joo et al.^19^ established a rapid UPLC-MS quantification method, enabling the simultaneous quantitation of three ginsenosides in 6.5 min of total run-time (i.e., the ginsenosides Re, Rg_1_, and Rh_1_ from ginseng berry extract). Qi et al.^21^ proposed a segmental monitoring and diagnostic ion filtering strategy in characterizing 70 ginseng saponins in ginseng root by rapid LC-TOF-MS. In our previous study, using a UPLC-QTOF-MS/MS technique we have also reported a method for simultaneously identifying 64 ginsenosides from ginseng flower^16^. In the present study, we explored effects of steaming at two different temperatures (100 or 120 °C) and for different durations (1, 2, 4 or 6 h) on ginsenosides composition of ginseng pulp utilizing the UPLC-QTOF-MS/MS technique and further quantified 18 representative ginsenosides using HPLC with the aid of their corresponding authentic standards. These exploitations would allow us to have a better understanding on ginsenosides composition profiles of the steamed and non-steamed ginseng pulps, and to canvass major structural changes of ginsenosides during steaming.

## Results and discussion

### Effects of steaming on HPLC profiles of ginsenosides in GPS and GPE

**Figure 1:**
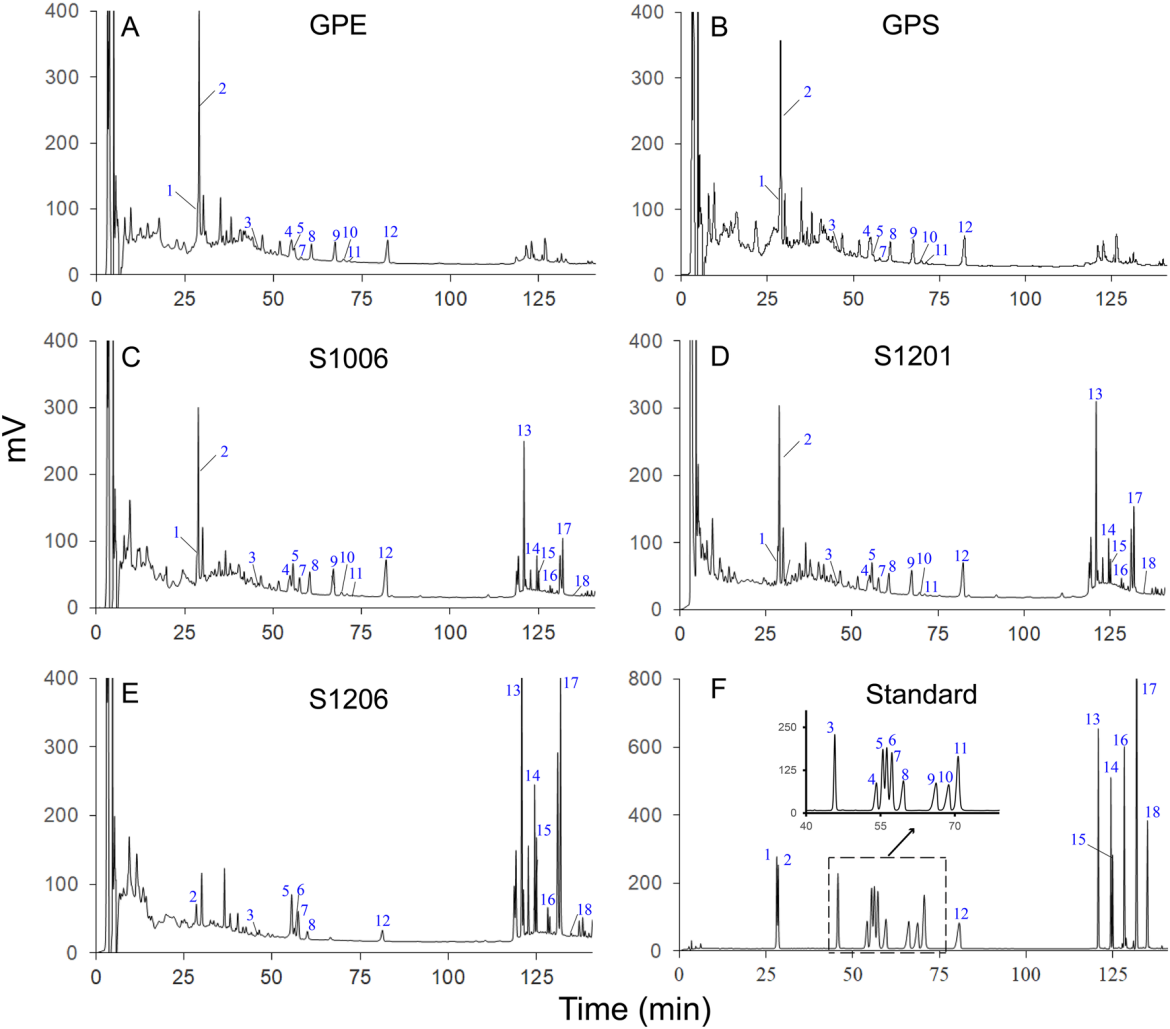
HPLC analyses of ginsenosides in extracts from raw and steamed ginseng pulps or ginseng pulp soaking supernatant. Shown here are chromatograms of (**A**) raw ginseng pulp extract (GPE); (**B**) ginseng pulp soaking supernatant extract (GPS); (**C**) GPS steamed for 6 h at 100 °C (S1006) and (**D**) for 1 h at 120 °C (S1201) and (**E**) GPS steamed for 6 h at 120 °C (S1206); and (**F**) standards of the 18 ginsenosides. (**F**) Ginsenosides marked are: (1) Rg_1_, (2) Re, (3) Rf, (4) Rb_1_, (5) 20(*S*)-Rg_2_, (6) 20(*S*)-Rh_1_, (7) 20(*R*)-Rg_2_, (8) Rc, (9) Rb_2_, (10) Rb_3_, (11) F_1_, (12) Rd, (13) F_4_, (14) 20(*S*)-Rg_3_, (15) 20(*R*)-Rg_3_, (16) 20(*S*)-PPT, (17) Rg_5_, and (18) Rh_2._ Peak numbers of the 18 standard ginsenosides that are not shown were not detected in the corresponding samples. An inset within (**F**) is an amplification of the partial profile between 40 and 85 min as indicated by the dashed box.

The HPLC method was used for characterizing profiles of ginsenosides in GPS and GPE before and after steaming. As shown in Fig. 1A,B, HPLC profiles of GPE and GPS before steaming present similar numbers of peaks at retention times between 25 and 90 min (polar ginsenosides) and between 110 and 140 min (less-polar ginsenosides), indicating that GPE and GPS contained similar species of ginsenosides, which suggests that 100% alcohol soaking of the ginseng berry enabled extraction of most ginsenosides contained in it. Notably, HPLC profile of GPS (Fig. 1B) exhibited a better separation effect than that of GPE (Fig. 1A); based on this fact, the effect of steaming on changes of ginsenoside compositions and individual ginsenoside levels were conducted with only GPS in ensuing experiments.

When GPS were steamed at 100 °C for 6 h (Fig. 1C), two opposite changes were observed in the profile of ginsenosides to that before steaming (Fig. 1B). That is, more species and contents of less-polar ginsenosides were detected between 110 and 140 min while levels of the individual polar ginsenosides between 25 and 90 min decreased slightly. When GPS were steamed at 120 °C for either 1 h (Fig. 1D) or 6 h (Fig. 1E), these trends of opposite changes became even obvious along with increasement of steaming temperature as well as elongation of the steaming duration: peak areas of the individual polar ginsenosides between 25 and 90 min further decreased, with some of them even disappeared; however those of less-polar ginsenosides between 110 and 140 were dramatically increased, especially those in GPS steamed at 120 °C for 6 h (Fig. 1E).

Overall, by comparing HPLC profiles in Fig. 1D,E with that in Fig. 1B,C, it is easy to figure out that high temperature and long duration of steaming were both essential for causing the opposite changes or transformations of polar into less-polar ginsenosides.

### Effects of steaming on transformation of 18 representative ginsenosides in GPE and GPS

#### Changes in total amount of the 18 ginsenosides during steaming

**Figure 2:**
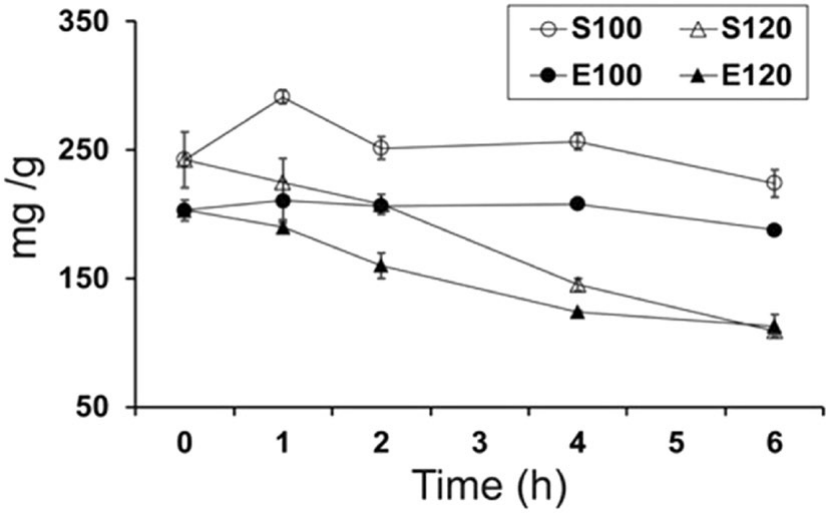
Changes in total contents of the 18 ginsenosides in raw and steamed ginseng pulps extracts (GPE) or extracts from ginseng pulp soaking supernatant (GPS) during steaming for 6 h at 100 °C (S100 or E100) and 120 °C (S120 or E100). Data at 0 h represent those of GPE or GPS. Values are denoted as the mean ± standard deviation (n = 3).

In order to quantitatively analyze the ginsenosides, we established a HPLC method that could simultaneously determine 18 representative ginsenosides. Figure 1F shows the HPLC profile of authentic standards of the 18 ginsenosides which were fine separated and could be unambiguously identified in each of the profiles (Fig. 1A–E). Total contents of these 18 ginsenosides were accordingly calculated for each of GPSs and GPEs steamed at 100 and 120 °C for 0–6 h (Fig. 2). As depicted in Fig. 2, it is clear that total contents of the 18 ginsenosides in GPS and GPE both slightly decreased after steaming. Especially, total contents of the 18 ginsenosides in GPS was higher than those in GPE, with the former being about 1.2 times higher than the later. With the extension of steaming duration at 100 °C, the total contents in GPS increased rapidly within 1 h, and restored to a stable level at 2–4 h, while the contents decreased gradually during the subsequent 4–6 h steaming. Similar to the trend of GPS, total contents of the 18 ginsenosides in GPE exhibited a relatively constant level within the 6 h, except the contents reduced slightly after 4 h. Notably, at 120 °C, total contents of the 18 ginsenosides of GPS and GPE decreased gradually along with the steaming time, both reaching their lowest values at 6 h.

Based on these results, it is obvious that steaming of GPE and GPS both resulted in decreases of total contents of the 18 ginsenosides. In terms of the total contents of the 18 ginsenosides, the optimal steaming temperature and time for both GPS and GPE were 100 °C 1 h (Fig. 2). However, as for the degree of transformation, GPS steamed at 120 °C for 6 h induced more thoroughly the conversion from polar to less-polar ginsenosides. Compared with our previous report on ginseng flowers upon baking and steaming^16^, the thermal stabilization of ginsenosides in GPE and GPS was even poorer. The long-term steaming at relatively low temperature (100 °C) caused the decomposition of ginsenosides, whereas higher temperature (120 °C) steaming made the decomposition even severer (Fig. 2). The above arguments may partly explain the decreases of ginsenosides content caused by long time of high temperature steaming, and the abundance as well as its poor heat stability of the polar ginsenoside Re in ginseng pulps, respectively, before and during steaming^22^. Besides, the higher ginsenosides contents in GPS than in GPE further indicates that most ginsenosides can be extracted by 100% ethanol soaking.

#### Transformation of low-active polar ginsenosides into high-active less-polar ginsenosides during steaming

**Figure 3:**
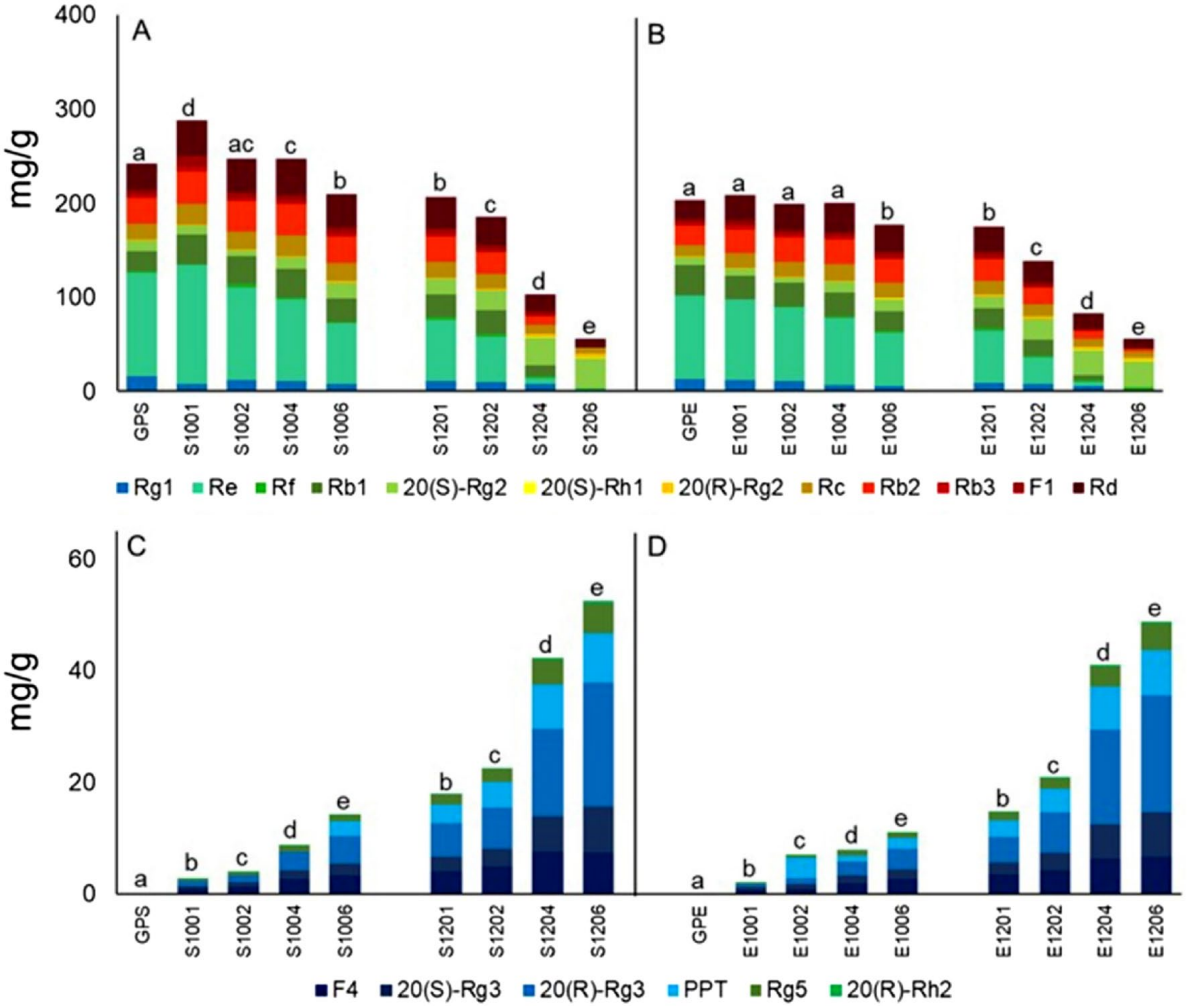
Changes in contents of the 18 ginsenosides in raw and steamed ginseng pulps extracts (GPE) or extracts from ginseng pulp soaking supernatant (GPS). Shown are contents of polar (**A**,**B**) and less-polar (**C**,**D**) ginsenosides, as indicated by differently-colored columns, in GPS (**A**,**C**) and GPE (**B**,**D**) steamed for 1–6 h at 100 °C (S100 and E100) and 120 °C (S120 and E120). Values marked by different letters at same temperatures are significantly different (*p* < 0.05) to those of GPE or GPS.

To further characterize transformation of ginsenosides during steaming, the 18 ginsenosides were divided into two groups. One includes 12 polar ginsenosides (Fig. 3A,B) which, occurred between 25 and 90 min in the HPLC profiles (Fig. 1F), are usually less bioactive in bioactivity. The other consists of six less-polar ginsenosides (Fig. 3C,D) which, occurred between 110 and 140 min (Fig. 1F), are usually more bioactive. It is worth noting that contents of individual ginsenosides were greatly different as indicated by the color gradient in an individual column of Fig. 3. One of the 12 polar ginsenosides, 20(*S*)-Rh_1_, was not detected in both GPS and GPE (Fig. 3A,B), and contents of the other 11 ranked as Re > Rd > Rb_2_ > Rb_1_ > Rc > Rg_1_ > 20(*S*)-Rg_2_ > F_1_ > Rb_3_ > Rf > 20(*R*)-Rg_2_, and Re > Rb_1_ > Rd > Rb_2_ > Rg_1_ > Rc > 20(*S*)-Rg_2_ > F_1_ > Rb_3_ > Rf > 20(*R*)-Rg_2_ (Fig. 3A,B), respectively. The six less-polar ginsenosides, which were also not detected in both GPS and GPE, gradually appeared during steaming and increased along with the enhancement of temperature and extension of duration. Obviously, total contents of the eleven polar and six less-polar ginsenosides were the highest in S1001 (Fig. 3A) and S1206 (Fig. 3C) among all the eight steaming treatments and they ranked in contents as Re > Rd > F_1_ > Rb_2_ > Rb_1_ > Rc > 20(*S*)-Rg_2_ > Rg_1_ > Rb_3_ > Rf > 20(*R*)-Rg_2_, and 20(*R*)-Rg_3_ > PPT > 20(*S*)-Rg_3_ > F_4_ > Rg_5_ > Rh_2_, respectively.

Considering total content of the 11 polar ginsenosides in GPS (Fig. 3A), it increased significantly at the 1st h then reduced significantly from the 4th to the 6th h of steaming at 100 °C, meanwhile it reduced significantly from the start during the 6 h steaming at 120 °C. On the other hand, total content of these 11 polar ginsenosides in GPE (Fig. 3B) exhibited no significant changes during the first 4 h then decreased significantly at the 6th h of steaming at 100 °C, meanwhile it decreased significantly from the start during the 6 h steaming at 120 °C.

Figure 3C,D show changes in total contents of less-polar ginsenosides in GPS and GPE during steaming at 100 and 120 °C, respectively. The six less-polar ginsenosides, which were not originally detected in both GPS and GPE, occurred during steaming. Their contents in GPS and GPE both increased from the 1st to the 6th h of steaming at 100 °C, and those steamed at 120 °C increased even dramatically, reaching the highest content at 120 °C for 6 h. Similar with polar ginsenosides, the whole tendency of less-polar ginsenosides in GPS still changed dramatically than that in GPE, and S1206 possessed the highest content of less-polar ginsenosides.

Above all, during the steaming processes of ginseng pulps, polar ginsenosides could be gradually converted to less-polar ginsenosides, resulting from raising of temperature (and/or pressure) and extending of steaming duration. Moreover, for the ginseng pulp, steaming treatment led to a significant reduction in content of polar ginsenosides, and the rapid increase of less-polar ginsenosides could not make up for this reduction due to the loss of glucoside ligands of polar ginsenosides and the degradation of certain species and/or amount of ginsenosides caused by continuous high temperature. Besides, GPS contained more polar or less-polar ginsenosides in various treatments compared to GPE.

### Identification of ginsenosides in raw and steamed ginseng pulps using UPLC-QTOF-MS/MS

As shown in Table 1, through analysing MS data and referring to available literatures as well as the MassBank MS database (http://www.massbank.jp/en/database.html), a total of 42 ginsenosides were identified: 18 (or 17 or 16) from GPS, 15 from GPE, and 31, 32, and 27 from S1006, S1201, and S1206, respectively, which were next classified into two groups, i.e., those originally existed in GPS and GPE (17 ginsenosides; data colored in black in Table 1) and those occurred during steaming (25 ginsenosides; data colored in blue). Obviously, more than half of the 42 ginsenosides were newly generated after steaming.

**Table 1:**
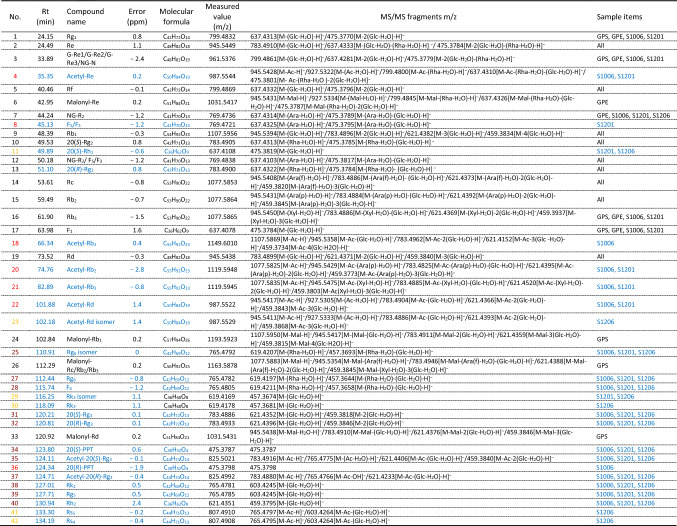
Identification of ginsenosides by UPLC-Q-TOF/MS in extracts from unsteamed and steamed ginseng pulps.

(i.e. Ara(f), α-L-arabinofuranose (150 Da); Ara(p), α-l-arabinopyranose (150 Da); Glc, β-d-glucopyranose (180 Da); Xyl, β-l-xylopyranose (150 Da); Rha, α-l-rhamnopyranose (164 Da); Mal, malonyl moiety (86 Da); Ac, acetyl moiety (42 Da). a. further confirmed in comparison with authentic standards. b. reported for the first time in steamed pulps.)

Data colored in black represent ginsenosides originally existed in extracts from fresh ginseng pulps (GPS and GPE), and those in blue represent ginsenosides newly generated in steamed extracts from ginseng pulps.

Numbers in the most left column colored in yellow represent ginsenosides newly generated in S1201 and/or S1206; those in brown, ginsenosides, newly generated in both S1006, S1201 and S1206; those in red, ginsenosides newly occurred in S1006 or S1201 but disappeared during high temperature and long period (S1206); and those in black, ginsenosides originally existed in GPS and/or GPE. GPS, extract from ginseng pulp supernatant; GPE, ginseng pulp extract; S1006, S1201 and S1206, steaming ginseng pulps at 100 °C for 1 h, at 120 °C for 1 h and 6 h, respectively.

#### Ginsenosides originally existed in GPS and GPE

Among the 20 ginsenosides originally existed in GPS and GPE, malonyl-Re (compound 6) detected from both GPS and GPE and malonyl-Rb_1_ (24), and malonyl-Rc or malonyl-Rb_2_ or malonyl-Rd (26), only from GPS, which have been previously reported^12,23^, all disappeared after steaming (Table 1). Malonyl ginsenosides were acidic ginsenosides existing in fresh ginseng, which possess the characteristics of high polar, strong hydrophilic and are easily soluble in water. It contains a malonyl residue attached to the glucose unit of the corresponding neutral ginsenoside, and the malonyl residue bond is very unstable, easily hydrolyzed and could cause hydrolysis reaction when meeting acid, alkali and hot water to generate corresponding neutral ginsenosides^21^. According to the follow-up results of this study, the abovementioned three of four malonyl ginsenosides identified (Table 1) were converted into their corresponding neutral ginsenosides [Re (compound 2), Rb_1_ (9), Rc (14) or Rb_2_(15), and Rd (19), respectively] during steaming.

The other 15 ginsenosides were identified as Rg_1_ (compound 1), Re (2), G-Re1 (or one of Re_2_, Re_3_, and NG-N) (3), Rf (5), NG-R_2_ (7), Rb_1_ (9), 20(*S*)-Rg_2_ (10), NG-R_2_/F_3_/F_5_ (12), 20(*R*)-Rg_2_ (13), Rc (14), Rb_2_ (15), Rb_3_ (16), F_1_ (17), and Rd (19), and malonyl-Rb_3_ (26) (Table 1, data in black). Most of them were abundant and common polar ginsenosides in GPS and GPE as well as in steamed ginseng pulps, indicating that they possessed relatively good thermostability and high content level. It should be noted that, different from the five malonyl ginsenosides described above, the fact that malonyl-Rb_3_ was still detected in the steamed ginseng pulp extract indicates that this special malonyl ginsenosides has high thermal stability or its initial formation content was high and had not been completely decomposed during steaming.

#### Ginsenosides newly generated in steamed ginseng pulps

The 25 ginsenosides newly generated in steamed GPS and GPE (Table 1, data colored in blue) could be further classified into the following three subgroups.

Six ginsenosides occurred solely after steaming at 120 °C for 1 h (S1201) and/or 6 h (S1206), and they were identified as 20(*S*)-Rh_1_ (compound 11), acetyl-Rd isomer (23), Rk_3_ isomer (29), Rk_3_ (30), Rs_5_ (41), and Rs_4_ (42) (Table 1, ginsenosides numbered in yellow color at the most left column). Among these six ginsenosides, two, i.e., 20(*S*)-Rh_1_ and Rk_3_ isomer (Table 1; Compounds 11 and 29), were detected both in S1201 and S1206, but the other four (Table 1; acetyl-Rd isomer, Rk_3_, Rs_5_, and Rs_4_), only in S1206, implying that 20(*S*)-Rh_1_ and Rk_3_ isomer possessed good thermal stability or higher content, while transformation of the other four ginsenosides required a relatively higher temperature, and might be the final product during steaming.

Seven ginsenosides newly occurred in S1006 or S1201 but disappeared during high temperature and long period (S1206), and they were identified as acetyl-Re (compound 4), F_5_/F_3_ (8), acetyl-Rb_1_ (18), acetyl-Rb_2_ (20), acetyl-Rb_3_ (21), acetyl-Rd (22), and 20(*R*)-PPT (36), respectively (Table 1, ginsenosides numbered in red at the most left column). Among them, acetyl-Rb_1_ (18) and 20(*R*)-PPT (36) appeared only in S1006, indicating that these two ginsenosides decomposed at 120 °C. F_5_/F_3_ was only detected in S1201 but neither in S1006 nor S1206, suggesting that although its occurrence needed higher steaming temperature (120 °C), extension of steaming time from 1 to 6 h finally led to its degradation. The other four ginsenosides (acetyl-Re, acetyl-Rb_2_, acetyl-Rb_3_, acetyl-Rd) were detected both from S1006 and S1201, indicating their poor stability at the prolonged higher temperature (pressures).

The other 11 ginsenosides occurred after all the three steaming treatments including those at 100 °C and 120 °C for 1 h (S1201) and 6 h (S1006 and S1206), and they were identified as Rg_6_ isomer (compound 25), Rg_6_ (27), F_4_ (28), 20(*S*)-Rg_3_ (31), 20(*R*)-Rg_3_ (32), 20(*S*)-PPT (34), acetyl-20(*S*)-Rg_3_ (35), acetyl-20(*R*)-Rg_3_ (37), Rk_1_ (38), Rg_5_ (39), and Rh_2_ (40) (Table 1; ginsenosides numbered in brown at the most left column). All of them were identified as less-polar ginsenosides, and appeared between 110 and 140 min (retention time), suggesting that steaming at higher temperature could lead to the transformation of polar ginsenosides to less-polar ginsenosides, and the latter were the finial products of ginsenosides conversion.

#### Identity assignment and confirmation of the ginsenosides in GPS, GPE and other steamed extracts of ginseng pulps

**Figure 4:**
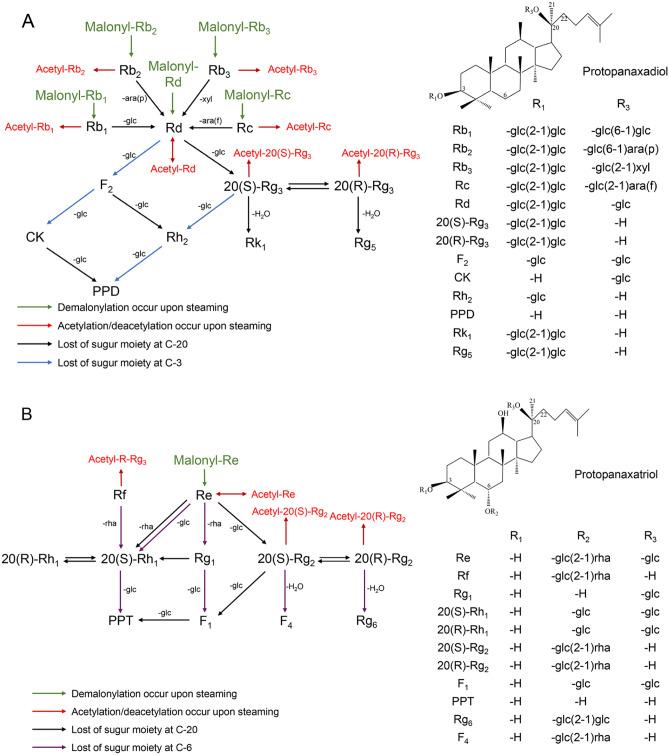
Chemical structures and possible transformation mechanisms of ginsenosides identified from raw and steamed ginseng pulps extracts (GPE) and/or extracts from ginseng pulp soaking supernatant (GPS). (**A**) Protopanaxadiols; (**B**) Protopanaxatriols. ara(f), α-l-arabinofuranosyl; ara(p), α-l-arabinopyranosyl; glc, β-d-glucopyranosyl; xyl, β-l-xylopyranosyl; rha, α-l-rhamnopyranosyl. Chemical links between C-20 and C-22 of F_4_ and Rg_5_, and between C-20 and C-21 of Rg_6_ and Rk_1_ are double bonds.

The 42 ginsenosides (Table 1) possess three features. Firstly, 33 could be classified as protopanaxadiol and protopanaxatriol ginsenosides due to their basic fragment ions at m/z of 459.38 and 475.37, respectively; except for 20(*R*)-PPT and 20(*S*)-PPT, all the other 40 involved in cleavage of a sugar moiety (or moieties) at C-3 and/or C-20 (protopanaxadiols) or at C-6 and/or C-20 (protopanaxatriols) from their corresponding protopanaxadiol or protopanaxatriol parent structure (for further information, see Fig. 4). The cleaved sugar moiety (or moieties) could be recognized by deducting a molecular mass of 162 (Glc-), 146 (Rha-), or 132 (Ara- or Xyl-) Da from the measured value listed in Table 1. Secondly, we identified four pairs of enantiomers, namely, 20(*R*)/20(*S*)-Rg_2_, 20(*R*)/20(*S*)-Rg_3_, 20(*R*)/20(*S*)-PPT, and 20(*R*)/20(*S*)-acetyl Rg_3_, based on the fact that retention time of an individual 20(*S*) ginsenoside was slightly shorter than that of its 20(*R*) ginsenoside^22,24^. Finally, 10 acetylated and six malonyl ginsenosides were identified via distinguishing losses of one or more 42-Da acetyl groups and 86-Da malonyl groups, respectively, from their corresponding molecular ion.

### Transformation mechanism of ginsenosides in ginseng pulps during steaming

Several reports characterized transformation mechanisms of ginsenosides during various processing of ginseng root^22,25^, and our previous report^16^ further demonstrated the underlying heat-induced chemical reactions in ginsenosides of ginseng flower. In this work, we for the first time quantified 18 representative ginsenosides in extracts from ginseng pulps at two typical temperatures (100 °C and 120 °C) of steaming for 1–6 h as described above. As generalized in Fig. 4, transformation of ginsenosides mainly involved four forms, namely, hydrolyzation, isomerization, acetylation, and demalonylation*.*

For protopanaxadiols (Fig. 4A), firstly, Rb_1_, Rb_2_, Rb_3_, Rc, and Rd could be converted by demalonylation from their corresponding malonyl-ginsenoside. Secondly, Rd could also be formed by hydrolyzing one glc-, ara(p)-, xyl- or ara(f)-residue attached to C-20 of Rb_1_, Rb_2_, Rb_3_ or Rc, then it was converted to F_2_ and 20(*S*)-Rg_3_ by further losing one glc-residue attached to its C-3 and C-20, respectively. Further hydrolyzing one more glc- at C-20 or C-3 of F_2_ would produce CK or Rh_2_, and at C-3 of 20(*S*)-Rg_3_, would only produce Rh_2_, and both CK and Rh_2_ were terminally converted to PPD by losing one glc-residue again. Thirdly, 20(*S*)-Rg_3_ could be isomerized to 20(*R*)-Rg_3_, and this pair of isomers could be further dehydrated at C-20 to yield Rk_1_ and Rg_5_, respectively. Fourthly, Rb_1_, Rb_2_, Rb_3_, Rc, 20(*S*)-Rg_3_, 20(*R*)-Rg_3_, and Rd could be acetylated to yield their corresponding acetyl-ginsenosides. In particular, acetylation and deacetylation of Rd were reversible.

For protopanaxatriols (Fig. 4B), similar to the transformation of Rb_1_, Rb_2_, Rb_3_ or Rc to Rd described above, changes of Re to 20(*S*)-Rh_1_, Rg_1_ and 20(*S*)-Rg_2_, Rf and Rg_1_ to 20(*S*)-Rh_1_ or 20(*R*)-Rh_1_, Rg_1_ and 20(*S*)-Rg_2_ to F_1_, and F_1_ and 20(*S*)-Rh_1_ to 20(*S*)-PPT occurred via hydrolyzing one sugar moiety or two as indicated. Furthermore, 20(*S*)-Rg_2_ and 20(*S*)-Rh_1_ could also be isomerized to 20(*R*)-Rg_2_ and 20(*R*)-Rh_1,_ respectively, of which 20(*S*)-isomers of Rg_2_ and 20(*R*)-Rg_2_ could become F_4_ and Rg_6_, respectively, by losing one H_2_O. Additionally, Rf, 20(*S*)-Rg_2_, 20(*R*)-Rg_2_, and Re could be acetylated to yield their corresponding acetyl-ginsenosides, and acetylation and deacetylation of Re were also reversible. Different from protopanaxadiols (Fig. 4A), for protopanaxatriols, only Re could be formed by demalonylating its corresponding malonyl-ginsenoside.

It has been proved that various fresh organs of ginseng plants possess unstable ginsenosides that are easily disappeared during a number of processing such as drying, baking, boiling, and steaming^1,13,14^, most of which are lower active polar ginsenosides and are in fact transformed into higher active less-polar ginsenosides via hydrolyzing and acetylation as abovementioned. For instances, by hydrolyzing of a series sugar moieties (Fig. 4), the polar Rd converted to less-polar F_2_ or 20(*S*)-Rg_3_, which would further produce less-polar CK, Rh_2_ and CK, and both CK and Rh_2_ were terminally converted to less-polar PPD; the polar Rf and Re could form the less-polar 20(*S*)-Rh_1_, which would further produce the less-polar PPT; and the polar Rg_1_ or 20(*S*)-Rg_2_ could yield the less-polar F_1_. Similar results have been previously reported in ginseng root, shoot, flower and berry or pulp^16,26,27^, but the types and transformation mechanisms of ginsenosides differed due to the different thermal conditions. For examples, Zhang et al.^26^ discovered that when ginseng root and shoot were steamed at 140 °C, the polar R1 was dehydrated to form the polar Rg_2_ under acid treatment which was further dehydrated to form the less-polar F_4_/Rg_6_ and then degraded into the less polar Rk_3_/Rh_4_.

The polar Rd was hydrolyzed to the less-polar Rg_3_ (S/R), which was then hydrolyzed to the less-polar Rg_5_/Rk_1_ and eventually degraded into the less polar Rk_2_/Rh_3_. When ginseng berry processed with microwave, polar Rb_2_ and Rf disappeared and polar Rb_1_ and Re decreased sharply, but less-polar Rg_3_ (S/R), Rh_4_, Rk_1_ and Rk_3_ appeared^27^.

Less-polar ginsenosides could also be reciprocally transformed via isomerization. For examples, the less polar pair of 20(*S*)-Rh_1_ and 20(*R*)-Rh_1_ and that of 20(*S*)-Rg_3_ and 20(*R*)-Rg_3_ could isomerize mutually, and the latter pair could be further dehydrated to yield less-polar Rk1 and Rg_5_, respectively. In addition, both the pair of polar 20(*S*)-Rg_2_ and 20(*R*)-Rg_2_ could transform to the less-polar F_4_ and Rg_6_, respectively. Different from previous studies in which only found one or two pairs of isomerized ginsenosides (mostly 20(*S*)-Rg_3_ and 20(*R*)-Rg_3_) were detected^14,21,26^, three pairs of isomerized ginsenosides were detected in the current our work, implying that our GPE and GPS from fresh ginseng pulps contained a considerable variety of ginsenosides.

The polarity of ginsenosides decreases when acetylated, thus less-polar ginsenosides could also be transformed by decarboxylation or acetylation; 10 less-polar acetyl-ginsenosides, namely, acetyl-Re, acetyl-Rb_1_, acetyl-Rb_2_, acetyl-Rb_3_, acetyl-Rd, acetyl-20(*S*)-Rg_3_, acetyl-20(*R*)-Rg_3_, Rs_5_, Rs_4_, and an acetyl-Rd isomer, were identified solely from steamed but not raw ginseng pulp extracts. In our previous study^16^, 23 less-polar acetyl-ginsenosides were detected in ginseng flower, but acetyl-Rb_1_, acetyl-Rs_5_, acetyl-Rs_4_, and an acetyl-Rd isomer were detected only in ginseng pulp. Xie et al.^25^ also reported two less-polar acetyl-ginsenosides, 20(*R*) acetyl-Re and acetyl-Rg_1_/isomer, in ginseng root steamed at 98 °C for 3 h. These results revealed that different acetyl-ginsenosides existed in different parts of the ginseng plant.

Yoon et al.^28^ detected malonyl-ginsenoside Rb1 and malonyl-ginsenoside Rd from seven cultivars of ginseng berry based on UPLC-QTOF/MS. Four malonyl-ginsenosides (malonyl-Re, malonyl-Rb_1_, malonyl-Rd, and malonyl-Rb_2_ or malonyl-Rb_3_ or malonyl-Rc), all of them are polar ginsenosides, were detected in GPS and GPE in the present study in that the ginseng pulps from which GPS and GPE were achieved were soaked in 100% ethanol immediately after picking. The results suggest the reason for various previous reports that did not detect malonyl-ginsenosides might be due to their ginseng materials were not fresh enough, from which all the malonyl-ginsenosides, except malonyl-Rb_3_, rapidly disappeared after harvesting. Similar results were reported by Xie et al.^25^, they identified 36 ginsenosides from red and white ginsengs and found the ratio of malonyl ginsenosides to their corresponding neutral ginsenosides in white ginseng ranged from 0.46 to 0.62 and from 0 to 0.19 in red ginseng. Qi et al.^21^ identified eight malonyl ginsenosides from fresh American ginseng and found that malonyl ginsenosides were more abundant in berries than in root.

## Methods

### Chemicals and preparation of crude extract from ginseng pulp

HPLC grade acetonitrile and other analytical grade reagents were purchased from Fisher Scientific (Pittsburgh, PA, USA) or Sinopharm Chemical Reagent Co. Ltd. (Beijing, China). Sixteen authentic standard ginsenosides [i.e., Rh_2_, 20(*S*)-Rg_2_, F_1_, Rd, Rb_1_, Rb_2_, Rf, 20(*S*)-Rg_1_, Rb_3_, Rc, 20(*R*)-Rh_1_, 20(*R*)-Rg_2_, 20(*S*)-Rg_3_, 20(*R*)-Rg_3_, 20(*S*)-PPT, and Re] were purchased from Lyle Biological (Luoyang, China), and two (F_4_ and Rg_5_) from Mansite Biological (Chengdu, China). Ultra-pure water was prepared using a milli-Q50 SP reagent water system (Millipore Corporation, Billerica, MA, USA).

Fresh ginseng berry of 3.0 kg, authenticated as the fruit of P. ginseng C.A. Meyer by Dr. Zhong-hua Liu (Beijing Forest University, China), was collected from Huanren county of Jilin province, China, soaked immediately in 5000 mL of 100% ethanol, and placed at room temperature for 5 days after bringing back to the laboratory.

Subsequently, the soaked berry was filtered using one layer of No.1 filter paper (Whatman, USA), with the filtrate (Filtrate I; roughly 5000 mL) containing compositions extracted from the pulp and the residues including the seed and the solid matter of the pulp. The residues, after removing the seed, were then ultrasonically extracted with 300 mL of 70% aqueous ethanol at 30 °C for 30 min for three times, and the three supernatants were combined and filtered also with one layer of the No.1 filter paper (Filtrate II; roughly 900 mL). Both filtrates I and II were rotarily evaporated and dried in a water bath at 40 °C to obtain crude extracts [i.e., extract from ginseng pulp soaking supernatant (GPS) and ginseng pulp extract (GPE) in respective] that was stored at − 20 °C until use.

### HPLC analyses for quantification of 18 representative ginsenosides

GPS or GPE of 3000 g d.w. in a vessel was steamed within an autoclave for 1, 2, 4 or 6 h at 100 °C (noted as S-1001, S-1002, S-1004 or E-1006; E-1001, E-1002, E-1004 or E-1006) or 120 °C (i.e., S-1201, S-1202, S-1204 or E-1206; E-1201, E-1202, E-1204 or E-1206). The sixteen steamed crude extracts and two unstreamed control extracts (i.e., GPS and GPE) were then stored at − 20 °C until HPLC analysis.

For quantitative determination of individual ginsenosides in each of the above 18 crude extracts, a stock solution of mixed authentic standards of the 18 ginsenosides as described above in subsection 2.1 was prepared and injected with six volumes (2, 4, 6, 10, 16 and 20 μL) for their linearity assessments, and good linearities within the range of 0.88–10.8 μg (*R*^2^ > 0.999; data not shown) and high precisions, stabilities and repeatabilities (with all their relative standard deviations being less than 5%) were obtained for each of the 18 ginsenosides.

HPLC analyses were performed using a Shimadzu HPLC system (Shimadzu, Japan) equipped with two LC-10AT VP pumps, a SPDM20A ultraviolet detector, and a SIL-20AC TH autosampler controlled by an analytical software (LC Solution-Release 1.23SP1). A reversed phase column (Diamonsil C18 5 μm 250 × 4.6 mm i.d., Dikma, Beijing, China) was used for separation, and the column temperature was set at 25 °C. The solvent system consisted of water (A) and acetonitrile (B) under the following gradient program: 0 min, 21% B; 0–14 min, 21% B; 14–24 min, 30% B; 24–55 min, 32% B; 55–75 min, 33% B; 75–100 min, 35% B; 100–120 min, 37% B; 120–130 min, 60% B; 130–140 min, 70% B; 140–150 min, 80% B. The flow rate was set at 0.8 mL/min with an injection volume of 20 µL. Detection wavelength was set at 203 nm to monitor more ginsenosides simultaneously^16^.

### UPLC-QTOF-MS/MS analyses for identification of ginsenosides

The UPLC-QTOF-MS/MS system was comprised of an Acquity Ultra-Performance Liquid Chromatography (UPLC) system and a Xevo G2-XS type QTOF-MS mass spectrometer (Waters, Milford, MA, USA). Each of the five selected crude extracts prepared above (GPS, S-1006, S-1201, S-1206 or GPE) was dissolved at a concentration of 1 mg/mL in chromatographic pure methanol, and the dissolved solution was filtered through a 0.22-μm membrane filter prior to the analysis with the UPLC under conditions exactly the same as that of the above HPLC, including the Diamonsil C_18_ column, solvent system and gradient program, flow rate, injection volume, and column temperature. Mass spectra were recorded within the range of m/z 100–1500 in both positive and negative ionization modes under the following conditions: nitrogen drying gas flow, 10.0 L/min; nebulizer pressure, 45 psi; gas drying temperature, 370 °C; capillary and fragment or voltage, 2500 kV; and MS/MS collision energies, 20 V^16^. The ginsenosides identified by negative ionization modes have covered those in positive modes, hence, the results were mainly based on negative ionization modes.

### Statistical analysis

All experimental results are expressed as means ± standard deviation (SD), and data were analyzed by one-way analysis of variance (*p* < 0.05) using SPSS software (ver. 17.0; SPSS Inc., Chicago, IL, USA).

## Conclusion

In the present study, we proved that all ginsenosides in ginseng pulps could be extracted by soaking in 100% ethanol, which might provide a new solution for the perishability of fresh ginseng pulp. In addition, to promote development of the abundant ginseng pulp resources, we for the first time clarified the ginsenoside transformation mechanisms under different temperatures (100 or 120 °C) for different durations (1–6 h), and found demalonylation and acetylation made the major contribution to the conversion of polar into less-polar ginsenosides. Four malonyl-ginsenosides (except for malonyl-ginsenoside Rb1 and malonyl-ginsenoside Rd) and ten acetyl-ginsenosides were simultaneously identified for the first time in extracts from ginseng pulp. These results might offer a new perspective for discovering novel compounds and popularizing this health-promoting functional foods.
